# The role of gut microbiota imbalance in preeclampsia pathogenesis: insights into FMO3-mediated inflammatory mechanisms

**DOI:** 10.3389/fmicb.2025.1682007

**Published:** 2025-12-12

**Authors:** Xia Xu, Ying Zhang, Yizheng Zu, Yanhong Xu, Tingting Liao, Xiuli Li, Jianying Yan

**Affiliations:** 1 College of Clinical Medicine for Obstetrics & Gynecology and Pediatrics, Fujian Medical University, Fujian Maternity and Child Health Hospital, Fuzhou, Fujian, China; 2 Fujian Clinical Research Center for Maternal-Fetal Medicine, Fuzhou, Fujian, China; 3 Laboratory of Maternal-Fetal Medicine, Fujian Maternity and Child Health Hospital, Fuzhou, Fujian, China; 4 National Key Obstetric Clinical Specialty Construction Institution of China, Fuzhou, Fujian, China; 5 The Second People's Hospital of Changzhou, The Third Affiliated Hospital of Nanjing Medical University, Changzhou, Jiangsu, China

**Keywords:** preeclampsia, FMO3, IL-8, gut microbiota imbalance, inflammatory reaction

## Abstract

**Background:**

Preeclampsia (PE) is a severe pregnancy complication linked to systemic inflammation and metabolic dysregulation. Emerging evidence suggests gut microbiota imbalance may contribute to PE pathogenesis, but the underlying mechanisms remain unclear. This study investigated whether gut dysbiosis triggers PE through flavin-containing monooxygenase 3 (FMO3)-mediated inflammatory pathways.

**Methods:**

We transplanted fecal microbiota from PE rats, healthy pregnant (HP) rats, and non-pregnant (NP) rats into antibiotic-treated dysbiotic rats, with a control group receiving normal saline (CON). Additionally, FMO3 expression was inhibited using FMO3-RNAi in parallel groups. We measured blood pressure, urine protein, FMO3 protein and mRNA expression, inflammatory markers, liver and kidney function, embryo resorption rate, and fetal weight. Gut microbiota composition was analyzed by 16S rRNA gene sequencing. The impact of interleukin-8 (IL-8) on trophoblast cell function was assessed using cell counting kit-8 (CCK-8), transwell invasion, and tube formation assays.

**Results:**

Rats receiving PE fecal microbiota transplantation (FMT) exhibited a gradual rise in blood pressure post-pregnancy, varying degrees of liver and kidney damage, markedly elevated serum inflammatory cytokines, higher fetal resorption rates, and reduced placental weights. FMO3 protein and mRNA expressions were significantly higher in the PE-FMT group. FMO3 knockdown partially improved these perinatal outcomes. Antibiotic treatment significantly decreased gut microbiota alpha and beta diversity. At the genus level, the PE-FMO3-RNAi group showed increased *Escherichia-Shigella* and decreased *Lactobacillus* compared to the PE-CON-RNAi group. In cell experiments, elevated IL-8 levels decreased the viability and invasiveness of HTR-8/SVneo cells and diminished the angiogenic potential of human umbilical vein endothelial cells (HUVECs).

**Conclusion:**

A disruption of gut microbiota could result in PE through the FMO3-driven inflammatory response, and targeting FMO3 may prove valuable in treating PE.

## 1 Introduction

Preeclampsia (PE) is a hypertensive disorder unique to pregnancy, characterized by new-onset hypertension and multi-organ dysfunction, contributing significantly to maternal and fetal morbidity and mortality (Dimitriadis et al., 2023; Sacks et al., 2018). While its exact pathogenesis remains incompletely elucidated (Opichka et al., 2021; Jung et al., 2022), PE is increasingly recognized as a state of systemic excessive inflammation. This is evidenced at the maternal-fetal interface, where inflammatory processes are closely associated with disease progression (Wang et al., 2022), and where women with PE exhibit elevated C-reactive protein, leukocyte activation, complement dysregulation, and platelet hyperactivity (Burwick and Feinberg, 2022; Jakobsen et al., 2019; Pierik et al., 2019; Suliman et al., 2023; Aggarwal et al., 2019). These inflammatory manifestations are consequences of placental dysfunction, which triggers a cytokine storm leading to widespread endothelial damage and vascular remodeling.

The gut microbiota, a key regulator of host metabolism and immunity (Ferraris et al., 2020; Al Bander et al., 2020; Fan and Pedersen, 2021; Afzaal et al., 2022), has emerged as a critical factor in various disease states, including pregnancy complications (Kim et al., 2019; Sultan et al., 2021; Hrncir, 2022; Pala et al., 2024). Dysbiosis has been linked to adverse gestational outcomes (Roth-Schulze et al., 2021) such as preeclampsia (Wang et al., 2024), gestational diabetes mellitus (GDM; Hasain et al., 2020), and preterm birth (Gershuni et al., 2021). For instance, maternal obesity and high-fat diets have been linked to gut dysbiosis and adverse neurodevelopmental outcomes in offspring, highlighting the intergenerational effects of maternal gut health (Di Gesù et al., 2022). Additionally, imbalances in gut microbiota have been linked to the development of GDM and preterm birth, both of which involve major metabolic and inflammatory disturbances (Ionescu et al., 2022; Balleza-Alejandri et al., 2024; Lu et al., 2022). These circumstances present immediate dangers to the health of both mother and baby, and they also carry lasting effects for both, such as heightened chances of heart diseases and metabolic issues (Rubini et al., 2022; Zhao et al., 2022; Xie et al., 2022).

Studies have shown that gut microbiota dysregulation can influence blood pressure through modifications of the intestinal epithelial barrier and microbial metabolites (Beale et al., 2019). Yang (2022) demonstrated maternal gut microbiota composition that may affect offspring gut microbiota. Beyond systemic inflammation, emerging evidence reveals that maternal dysbiosis can directly disrupt placental fuel metabolism. For example, Giugliano et al. (2025) demonstrated that gut microbiota dysbiosis impairs placental carbohydrate metabolism, an effect that can be rescued by glucose supplementation and the placenta's own metabolic programming is now recognized as a central determinant of pregnancy outcomes (Chen et al., 2022). One proposed pathway involves microbiota-driven systemic inflammation, characterized by elevated inflammatory cytokines including interleukin-6 (IL-6), interleukin-1 beta (IL-1β), and tumor necrosis factor-alpha (TNF-α; Yong et al., 2022). Supporting this, probiotic interventions in PE models have shown benefits in reducing endotoxemia, stabilizing microbial communities, improving endothelial function, and reducing hypertension and inflammation (Sun et al., 2020).

A particularly relevant microbial metabolite is trimethylamine-N-oxide (TMAO), generated from dietary precursors through the action of gut microbial enzymes and subsequent hepatic conversion by flavin-containing monooxygenase 3 (FMO3; Luo et al., 2024; Chen et al., 2019; Iglesias-Carres et al., 2023; Yang et al., 2022). This enzyme facilitates the N-oxygenation of TMA, producing non-smelly trimethylamine N-oxide (TMAO), a crucial reaction to avoid the buildup of foul-smelling TMA, responsible for trimethylaminuria or fish odor syndrome (Gatarek and Kaluzna-Czaplinska, 2021; Liang et al., 2020). Extensive research on genetic variations in the FMO3 gene has shown considerable differences in enzyme activity among individuals (Nguyen et al., 2019; Phillips and Shephard, 2020). These polymorphisms can lead to altered enzyme function, impacting drug metabolism and susceptibility to adverse drug reactions. FMO3 expression is highly variable among individuals and can be influenced by genetic factors rather than environmental ones (Nguyen et al., 2019). Additionally, hormonal changes, such as those related to menstruation, can modulate FMO3 expression levels, further contributing to interindividual variability (Esposito et al., 2014). Various quantitative clinical and experimental data indicate that dietary fats can influence TMAO levels via gut bacteria or FMO3 enzyme function (He et al., 2020). Koh et al. (2019) found through animal experiments that pterostilbene could partially alleviate carnitine induced vascular inflammation by regulating gut microbiota and reducing FMO3 levels.

These study findings indicate a strong connection between FMO3, gut microbiota, and the body's overall inflammatory response. This research seeks to determine if disruptions in gut microbiota can influence the development of PE and if such disturbances contribute to PE pathogenesis via an FMO3-driven inflammatory response.

## 2 Materials and methods

### 2.1 Establishment of a rat model of PE

On the 9th day of pregnancy, Sprague-Dawley rats in the PE-FTD (Preeclampsia-feces transplant donor) group were subcutaneously injected with N-nitro-arginine methyl Ester (L-NAME, 75 mg/kg/d) to prepare a preeclampsia model until 17th day of pregnancy (Shu et al., 2018), and rats in the HP-FTD group (Healthy pregnant—feces transplant donor) were injected with an equal volume of normal saline. On the 18th day of pregnancy, the systolic blood pressure (SP) and urine protein were detected. If the blood pressure and urine protein of rats in the PE-FTD group increased significantly, it indicated that the preeclampsia model was successfully established. On the 18th day of pregnancy, the feces were collected by sterile fecal collector.

### 2.2 Donor fecal bacteria treatment

The fecal transplant donors included healthy sexually-mature non-pregnant female Sprague-Dawley (SD) rats, PE rats, and full-term pregnant rats. Fecal liquid treatment was prepared as follows: we used a sterile fecal collector to obtain an appropriate amount of fresh feces from the donors. This was sealed in a biological ice bag and transferred to the operating room for immediate processing. A feces slurry was made by transferring 6 pieces of rat feces to a sterile 50 mL centrifuge tube, adding 8 mL of sterile normal saline, and vortexing the mixture for 10 min at 1,000 rpm. The mixture was filtered using a sequence of sieves with pore sizes of 2.0 mm, 0.707 mm, 0.27 mm, and 0.15 mm. The filtrate was centrifuged at 6,000 g at 4 °C for 15 min. The sediment was dissolved again in 5 milliliters of sterile normal saline. The fecal suspension was added with 10 % sterile glycerol. All steps were performed on ice when possible. The feces were stored in an ultra-low temperature refrigerator at −80 °C.

### 2.3 Establishment of a rat model of flora imbalance

Female and male SD rats were acquired from Shanghai SLAC Laboratory Animal Co., Ltd. When they were 8 weeks old, a combination of oral antibiotics (neomycin 1 g/L, vancomycin 500 mg/L, ampicillin 1 g/L, metronidazole 1 g/L) was given for 5 days to suppress gut bacteria via drinking water. Each female SD rats mated with one male SD rats. Then we collected feces from the animals and characterized the abundance of gut microbiota species to prove the successful establishment of animal model of gut microbiota imbalance.

### 2.4 Fecal bacteria transplantation

Following antibiotic treatment, each rat was fed with 1 mL of the appropriate gut microbiota via gavage (once a day for 3 days and 3 times a week until the 17th day of pregnancy). Animals were assigned to four groups: antibiotic + normal saline group (CON), antibiotic + PE rat feces group (PE), antibiotics + health pregnant rat feces group (HP) and antibiotic + non-pregnant rat feces group (NP; *n* = 5 in each group).

Moreover, to explore the role of FMO3 in PE, CON-RNAi and FMO3-RNAi adenoviruses (5 × 109 PFU in 100 μL) were injected into the tail vein when rats firstly received the antibiotic treatment and once 3 weeks until the 17th day of pregnancy. The rats were divided into 6 groups (*n* = 5 in each group) which including: antibiotic + PE rat fecal CON-RNAi (PE-CON-RNAi), antibiotic + health pregnant rat fecal CON-RNAi (HP-CON-RNAi), antibiotic + non-pregnant rat fecal CON-RNAi (NP-CON-RNAi), antibiotic + PE rat fecal FMO3-RNAi (PE-FMO3-RNAi), antibiotic + health pregnant rat fecal FMO3-RNAi (HP-FMO3-RNAi), antibiotic + non-pregnant rat feces FMO3-RNAi (NP-FMO3-RNAi). After 6 weeks of fecal microbiota transplantation (FMT), male and female rats were caged at a ratio of 1:1. The vaginal plug was marked as day 0 of pregnancy (E0).

Urine protein was measured once before FMT, blood pressure was measured weekly after FMT, urine protein was measured once before mating, and blood pressure was measured every 3 days after mating. Urine protein in 24 h was collected in 17th of day of pregnancy. On the 18th day of pregnancy, the rats were dissected to obtain serum, liver, kidney, placenta, fetus and other samples.

### 2.5 Gut microbiota processing and sequencing

The total DNA of fecal samples was extracted. According to the E.Z.N.A.^®^ Stool DNA Kit instructions, total DNA extraction was performed. The extracted DNA was stored in a refrigerator at −20 °C. The V3–V4 variable region of bacterial 16S rDNA gene was selected for sequencing. To select the region for PCR amplification, use bacterial universal primers: Upstream primer: 5′-ACTCCTACGGGGAGGCAGCAG3′; Downstream primer: 5′ -GGACTACHVGGGTWTCTAAT-3′. It is purified according to the standard operating procedures of the Illumina MiSeq platform. The amplified PCR fragments were used to construct the library. Miseq PE300 sequencer was used for sequencing. The diversity index, community abundance and sequencing coverage of the sample flora were analyzed.

Alpha diversity analysis, Beta diversity analysis, species composition analysis, and species difference analysis were performed on microorganisms. Mothur software (version v.1.30) was used to evaluate the Alpha diversity index of the samples. Alpha diversity analysis can reflect the abundance and diversity of microbial communities in the samples, including chao1, Simpson, dominance, Good's coverage, Observed otus, Pielou e and Shannon methods. Chao1 index can reflect the microbial richness of the sample and estimate the number of species actually present in the community. Shannon index and Simpson index can reflect the microbial diversity of the samples. Principal coordinate analysis (PCo A) of Beta diversity was performed using Qiime software (V1.9.1; Principal coordinate analysis is a non-binding data dimension reduction analysis method that can study the similarity or difference of sample community composition. Different points represent different samples. The distance between samples represents the similarity between samples. The closer the distance, the greater the similarity). The results are visualized by R language. For statistical analysis of microbiota data, non-parametric tests were employed due to the non-normal distribution characteristics of microbiome data. For comparisons of alpha diversity indices among multiple groups, the Kruskal-Wallis test was used, followed by Dunn's *post-hoc* test. For comparisons of species abundance between two groups, the Wilcoxon rank-sum test was applied. Beta diversity was assessed using Permutational Multivariate Analysis of Variance (Adonis/PERMANOVA) to test for differences in community structure between groups.

### 2.6 Blood pressure measurement

BP was measured when the animals were awake by non-invasive caudal artery pulse manometry. Measurement was conducted as follows. The equipment was connected and the sphygmomanometer was calibrated. When the temperature of equipment reached 39 °C, the animal was immobilized and the pressure sensor was placed on the base of the tail, measurement commenced after the pulse became stable but the whole process must be completed within 10 min. The SP of each rat was measured ≥6 times (Valid data: The SP of the rats with the smallest average difference in three consecutive resting states, and the difference between the three results was within 10 mmHg.). The mean SP value was then recorded for the animal.

### 2.7 Western blotting analysis

Protein lysate was generated by grinding the liver samples with RIPA buffer and PMSF and then left on ice for 20 min. After centrifugation at 12,000 rpm for 20 min at 4 °C, 400 μL of the supernatant was transferred to an eppendorf tube. The protein levels in the samples were measured using a BCA assay. After the protein samples were diluted to the same concentration using RIPA, 100 μL of the sample and 20 μL of loading buffer were thoroughly mixed together. The samples were immersed in a 100 °C -water bath for 5 min before being stored at −80 °C. The specimens were isolated via SDS-PAGE in reducing environments, then moved to a PVDF membrane (Millipore, Massachusetts, USA). The PVDF membranes, after being transferred, were blocked using 5% skim milk on a shaker set to 65 rpm for 1 h at room temperature. Subsequently, they were incubated with primary antibodies FMO3 (ab126711, Abcam, Cambridge, UK, 1:1000) and β-actin (ab8226, Abcam, Cambridge, UK, 1:1000) overnight at 4 °C on a shaker. The PVDF membranes underwent three 10-min washes with TBST. Following the wash, the PVDF membrane was immersed in a secondary antibody solution (1:5000) and incubated for 2 h at ambient temperature on a shaker. Subsequently, the PVDF membranes underwent three washes with TBST, each lasting 10 min. Following another wash, the proteins tagged with antibodies were identified through chemiluminescence using the Sensitive ECL Luminescence Reagent (MA0187, Meilunbio, Dalian, China) within the Versa DocTM Imaging System. β-actin served as a control to determine the relative amounts of the target protein.

### 2.8 Quantitative real-time PCR

Liver tissue RNA was extracted with Trizol Reagent (Invitrogen, Carlsbad, USA). Following the quantification of RNA levels, 1 μg of RNA was converted into complementary cDNA utilizing the cDNA Synthesis SuperMix from NovoProtein (Suzhou, China). Following the manufacturer's instructions, the cDNA template was utilized with NovoStart SYBR qPCR SuperMix Plus (NovoProtein, Suzhou, China) on the QuantStudio 3 Real-Time PCR System (Thermo Fisher Scientific, Waltham, USA) for qRT-PCR. The expression levels of the target gene were standardized using β-actin as a reference control through the 2–ΔΔCT technique. Table 1 displays the primer sequences that were utilized.

**Table 1:** Primer sequences of genes.

**Gene**	**Forward primer**	**Reverse primer**
FMO3-shRNA1	5′ATCCGCATAACAGCAAGCTCCAAGATTCAAGAGATCTTGGA GCTTGCTGTTATGCTTTTTG3′	5′GGATCAAAAAGCATAACAGCAAGCTCCAAGATCTCTTGAAT CTTGGAGCTTGCTGTTATGCG3′
FMO3-shRNA2	5′GATCCGCTTCCACAGCAGGGACTATATTCAAGAGATATAGT CCCTGCTGTGGAAGCTTTTTG3′	5′GGATCAAAAAGCTTCCACAGCAGGGACTATATCTCTTGAAT ATAGTCCCTGCTGTGGAAGCG3′
FMO3-shRNA3	5′GATCCGGAAGGAGCCTGTGTTCAATGTTCAAGAGACATTG AACACAGGCTCCTTCCTTTTTG3′	5′GGATCAAAAAGGAAGGAGCCTGTGTTCAATGTCTCTTGAAC ATTGAACACAGGCTCCTTCCG3′
β-actin	5′CGCGAGTACAACCTTCTTGC3′	5′CCTTCTGACCCATACCCACC3′

### 2.9 Liver and kidney HE staining

The sections were dried through a series of immersions: 20 min in xylene I, 20 min in xylene II, 5 min in absolute ethanol I, 5 min in absolute ethanol II, and 5 min in 75% alcohol, followed by a rinse with tap water. The samples were immersed in hematoxylin dye for 3–5 min and subsequently rinsed with tap water. The segments were immersed in the differentiation medium, rinsed with tap water, treated with reverse-blue solution, and finally washed with running water. The sections were then successively dehydrated in 85% and 95% alcohol for 5 min and then stained in eosin staining solution for 5 min. The segments were sequentially immersed in absolute ethanol I for 5 min, absolute ethanol II for 5 min, absolute ethanol III for 5 min, dimethyl I for 5 min, and xylene II for 5 min to clear them, and then sealed with neutral gum. The sections were examined under a microscope and images were collected for analysis.

### 2.10 Concentrations of TMAO, IL-1β, IL-8, and TNF-α in the blood

The concentrations of IL-1β, IL-8, and TNF-α were measured with Rat IL-1β, IL-8, and TNF-α ELISA kits, all from USCN Life, WuHan, China. The concentrations of TMAO were measured with Rat TMAO ELISA kits (MEIMIAN, JiangSu, China). Every ELISA test was conducted following the guidelines provided by the manufacturers, ensuring rigorous quality control protocols were adhered.

### 2.11 Cell culture

HTR-8/SVneo cells were obtained from Meisen CTCC (Meisen Chinese Tissue Culture Collections) and cultured at 37 °C with 5% CO_2_ in 1640 medium (Gibco, New York, NY, USA) supplemented with 1% penicillin-streptomycin and 10% fetal bovine serum. Human Umbilical Vein Endothelial Cells (HUVECs) obtained from Meisen CTCC (Meisen Chinese Tissue Culture Collections) were cultured in an incubator with 5% CO_2_ at 37 °C, using F12K medium supplemented with 1% penicillin-streptomycin, 10% fetal bovine serum, 0.1 mg/mL heparin, and 30 μg/mL Endothelial Cell Growth Supplement (ECGS). Media was changed every 1–2 days, and the cells were passaged every 2–3 days.

### 2.12 Cell viability assay

For the treatment, IL-8 was prepared at concentrations of 0, 10, 100, and 1,000 pg/mL. A suspension of HTR-8/SVneo cells at a concentration of 4 × 10^4^ cells/mL was made, and 100 μL was distributed into each well of a 96-well plate, with three replicates for each IL-8 concentration. HTR-8/SVneo cell viability was assessed with the Cell Counting Kit-8 from TransGen Biotechnology (Beijing, China) at intervals of 0, 24, and 48 h. A 10% CCK-8 solution was introduced to each well and incubated at 37 °C for 2 h. Subsequently, the optical density (OD) readings at 450 nm were recorded using an enzyme labeling device from Thermo Fisher Scientific, Waltham, MA, USA.

### 2.13 Invasion assay

Matrigel was mixed with serum-free medium at a 1:8 ratio on ice, and 50 μL was subsequently used to coat the upper chamber of the Transwell (Corning, NY, USA). This was air dried at 4 °C and solidified thoroughly at 37 °C. For every group, we introduced 100 μL of a cell suspension containing 1 × 10^5^ cells/mL into the upper section of the prepared Transwell chamber, followed by incubation in plates containing medium with 10% FBS. Cells in the IL-8 + Repertaxin group were pretreated with 10 μM Repertaxin for 2 h and 100 pg/mL IL-8. After 24 h, the Transwell chambers were removed and the cells of the remaining matrigel were wiped with cotton swabs. The cells underwent three PBS washes, were subsequently fixed using paraformaldehyde, stained with crystal violet, and finally counted under a microscope.

### 2.14 Tube-formation assay

Tube formation tests in Matrigel (Corning, NY, USA) were used to evaluate *in vitro* angiogenesis. Each well of a 96-well plate received 50 μL of matrigel on ice, which was then solidified at 37 °C for 1 h Cells in the IL-8 + Repertaxin group were pretreated with 10 μM Repertaxin for 2 h and 100 pg/mL IL-8. After treating the HTR8/sVneo cells, they were placed into 96-well plates at a concentration of 3 × 10^4^ cells and incubated for 8 h. The impact on endothelial tube formation was then examined using an inverted microscope. The randomly selected fields were captured.

### 2.15 Statistical analysis

All data were analyzed by GraphPad Prism 9.5 and R. The data is presented as the average value plus or minus the standard deviation (mean ± SD). One-way analyses of variance were used to compare the differences among multiple groups. *T* tests were used to compare the differences between two groups. For microbiota data analysis, non-parametric tests were used including Wilcoxon rank-sum test for two-group comparisons and Kruskal-Wallis test with Dunn's *post-hoc* test for multiple group comparisons, considering the non-normal distribution characteristics of microbiome data. The statistical threshold was set at α = 0.05.

## 3 Results

### 3.1 Preeclampsia SD rat model was successfully established

**Figure 1:**
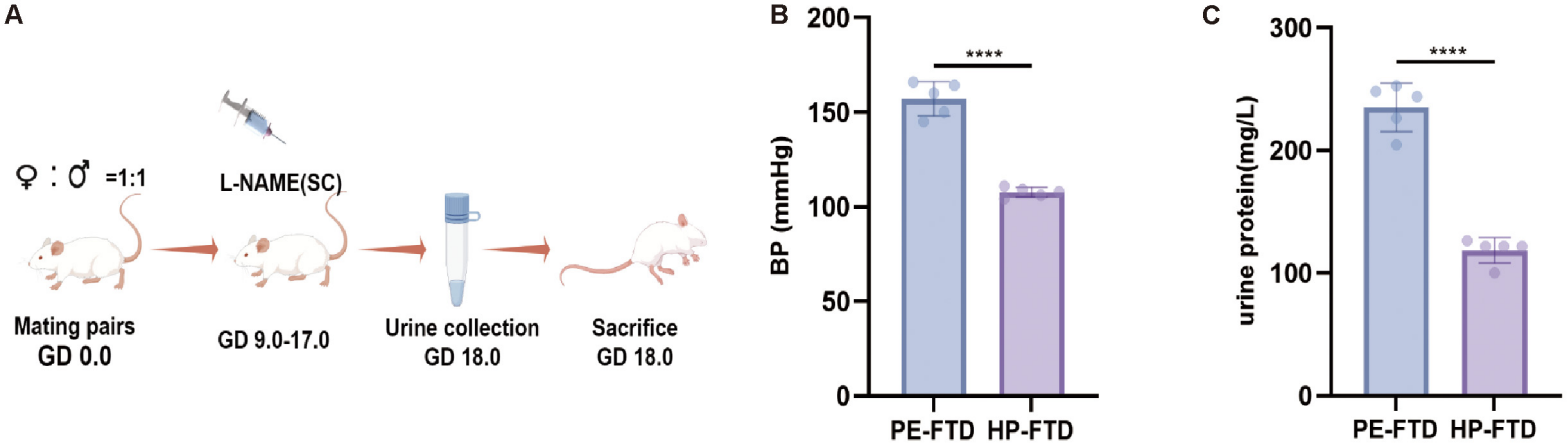
Characterization of the preeclampsia-like rat model. **(A)** Preeclampsia-like SD rats model establishment process. **(B)** Changes in blood pressure between PE-FTD (Preeclampsia -feces transplant donor) group and HP-FTD (Healthy pregnant-feces transplant donor) group. **(C)** Changes in urine protein between PE-FTD group and HP-FTD group (PS: ****p < 0.0001, data were analyzed by *T* tests).

PE-FTD (Preeclampsia-feces transplant donor) group were subcutaneously injected with L-NAME (75 mg/kg/d) for 9 days to establish a preeclampsia-like SD rat model, and rats in the HP-FTD (Healthy pregnant-feces transplant donor) group were injected with an equal volume of normal saline. Their blood pressure and urine protein were measured on the 18th day of pregnancy. All processes were showed in Figure 1A. It was found that the blood pressure and urine protein in PE-FTD group were significantly increased (Figures 1B, C), which was consistent with the manifestations of preeclampsia.

### 3.2 PE-fecal microbiota transplantation in SD rats can induce PE-like symptoms

**Figure 2:**
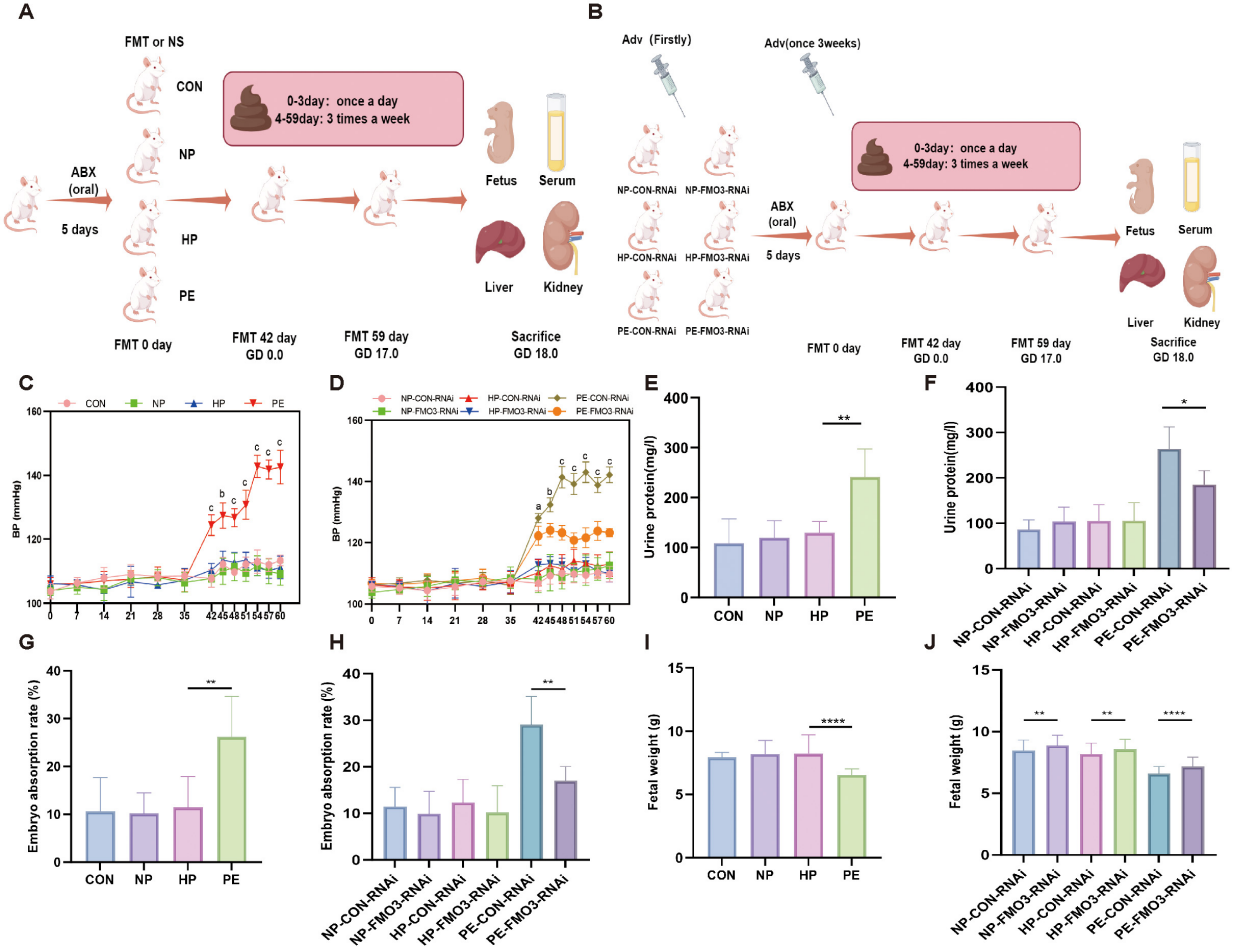
Maternal blood pressure and fetal and placental growth and development. **(A)** Fecal microbiota transplantation (FMT) processes. **(B)** FMO3-RNAi adenovirus infected rats model establishment process. **(C)** Blood pressure changes in FMT rats. **(D)** Blood pressure changes in FMT rats injected with FMO3-RNAi adenovirus. **(E)** Urine protein changes in FMT rats. **(F)** Urine protein changes in FMT rats injected with FMO3-RNAi adenovirus. **(G)** Embryo absorption rate (the percentage of fetal loss which was calculated as following: % of resorption = R/(R + V) × 100, where R represents the number of hemorrhagic implantation (sites of fetal loss) and V stands for the number of viable, surviving fetuses.) in FMT groups. **(H)** Embryo absorption rate after FMO3-RNAi adenovirus injection in FMT groups. **(I)** Fetal weight in FMT groups. **(J)** Fetal weight after FMO3-RNAi adenovirus injection in FMT groups (PS: ^a^*p* < 0.05, ^b^*p* < 0.01, ^c^*p* < 0.0001, *p < 0.05, **p < 0.01, ****p < 0.0001, data were analyzed by one-way ANOVA).

In order to explore the relationship between gut microbiota and preeclampsia, we transplanted the feces of PE rats modeled by L-NAME into SD rats treated with antibiotics (Figure 2A). As a control, we also set up normal saline (CON), health pregnant (HP) and non-pregnant (NP) rat fecal microbiota transplantation groups at the same time.

The results indicated that the systolic blood pressure in the CON, NP, and HP groups remained stable throughout the fecal bacteria transplantation in rats, with no significant differences observed between pregnant and non-pregnant states. However, the PE group exhibited a notable rise in systolic blood pressure from the first day to the 18th day of pregnancy (FMT 42–60 days, Figure 2C). Meanwhile, the urine protein of PE group also exhibited a notable rise on 17th day of pregnancy (Figure 2E).

Moreover, the study on fetal rats and placental development revealed that the embryo absorption rate (the percentage of fetal loss which was calculated as following: % of resorption = R/(R + V) × 100, where R represents the number of hemorrhagic implantation (sites of fetal loss) and V stands for the number of viable, surviving fetuses.) in the PE group was significantly higher than in the HP group (*p* < 0.01). However, there was no statistical difference in embryo absorption rates among the CON, NP, and HP groups, as illustrated in Figure 2G. The fetal weight of PE group was obviously less than HP group (*p* < 0.0001), while the CON, NP and HP group had no statistical difference (Figure 2I). These results indicate that typical preeclampsia-like symptoms can occur in the PE group after PE-FMT.

### 3.3 FMO3 level increased in PE group and FMO3 knockdown alleviated FMT-induced preeclampsia symptoms in rats

**Figure 3:**
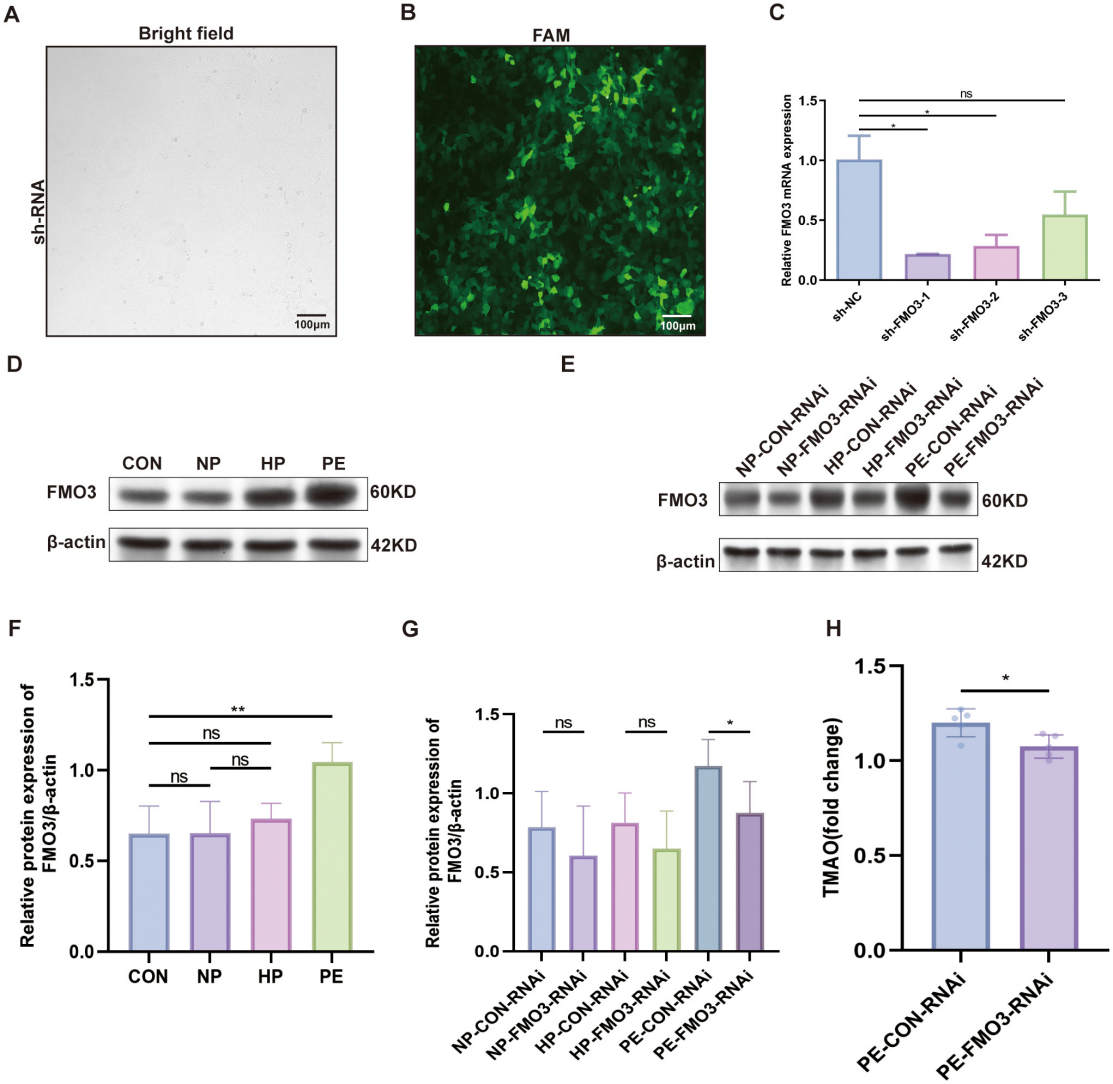
FMO3-RNAi adenovirus infection and validation. **(A, B)** Fluorescence plot of FMO3-RNAi adenovirus transfection in HEK 293A cells. **(C)** qRT-PCR confirmed the effectiveness of the FMO3 knockdown, leading to the selection of FMO3-shRNA for further studies. **(D, E)** Western blotting was employed to assess the FMO3 protein concentrations. **(F, G)** Protein quantities of FMO3 across various groups were assessed through Western blotting following infection with FMO3 adenovirus. **(H)** Comparison of serum TAMO levels in SD rats after injection of FMO3 adenovirus in PE group (PS: *p < 0.05, **p < 0.01, all the results based on at least three biological and technical repetitions, data of multiple groups were analyzed by one-way ANOVA. *T* tests were used to compare the differences between two groups).

In order to clarify the relationship between PE and fecal microbiota, we detected the FMO3 protein level in the PE group and found that it was significantly increased (Figures 3D, F). Then FMO3-RNAi adenovirus were used in FMT rats to explore whether FMO3 played an important role in this process (Figure 2B). Firstly, we checked the infection and knockdown efficiencies of FMO3-RNAi (Figures 3A–C, E, G). We found that after control adenovirus injection, the FMO3 level of each control group showed similar results as before, and the level of PE-CON-RNAi group was significantly increased (Figures 3E, G).

We also observed the blood pressure, urine protein, embryo resorption rate and fetal weight in these groups. NP-CON-RNAi, NP-FMO3-RNAi, HP-CON-RNAi and HP-FMO3-RNAi group of rats during the entire fecal bacteria transplanted systolic blood pressure is stable, and there was no significant difference between pregnant and non-pregnant rats. However, Systolic blood pressure of PE-CON-RNAi group rats significantly rose in whole pregnancy. The result in the PE-FMO3-RNAi group suggested that this abnormal systolic blood pressure increase during pregnancy would be alleviated after injecting FMO3-RNAi adenovirus, as shown in Figure 2D.

At same time, results of urine protein, embryo resorption rate and fetal weight suggest the same conclusion. The PE-CON-RNAi group exhibited a notably higher urine protein (*p* < 0.05) and embryo resorption (*p* < 0.01) compared to the PE-FMO3-RNAi group (Figures 2F, H). The weight of fetuses in the PE-CON-RNAi group was markedly less compared to those in the PE-FMO3-RNAi group (*p* < 0.0001; Figure 2J). As illustrated in Figure 2J, the fetal weight in the NP-CON-RNAi group was markedly less than that in the NP-FMO3-RNAi group (*p* < 0.0001), and similarly, the HP-CON-RNAi group exhibited a significantly lower fetal weight compared to the HP-FMO3-RNAi group (*p* < 0.0001). However, there was no significant difference in urinary protein and embryo resorption rate of HP and NP groups before and after injection of FMO3 adenovirus. Furthermore, we detected the serum TMAO level, and found that after knocking down FMO3, the TMAO level of PE group was significantly reduced (Figure 3H). The above results indicate that gut microbiota may involve in the pathogenesis of preeclampsia and the abnormal increase of FMO3 may be involved in this pathological process. Knockdown of FMO3 has a significant alleviating effect on preeclampsia-like symptoms.

### 3.4 PE-fecal microbiota transplantation in SD rats can lead to PE-like pathological damage of liver and kidney which were alleviated by FMO3 knockdown

**Figure 4:**
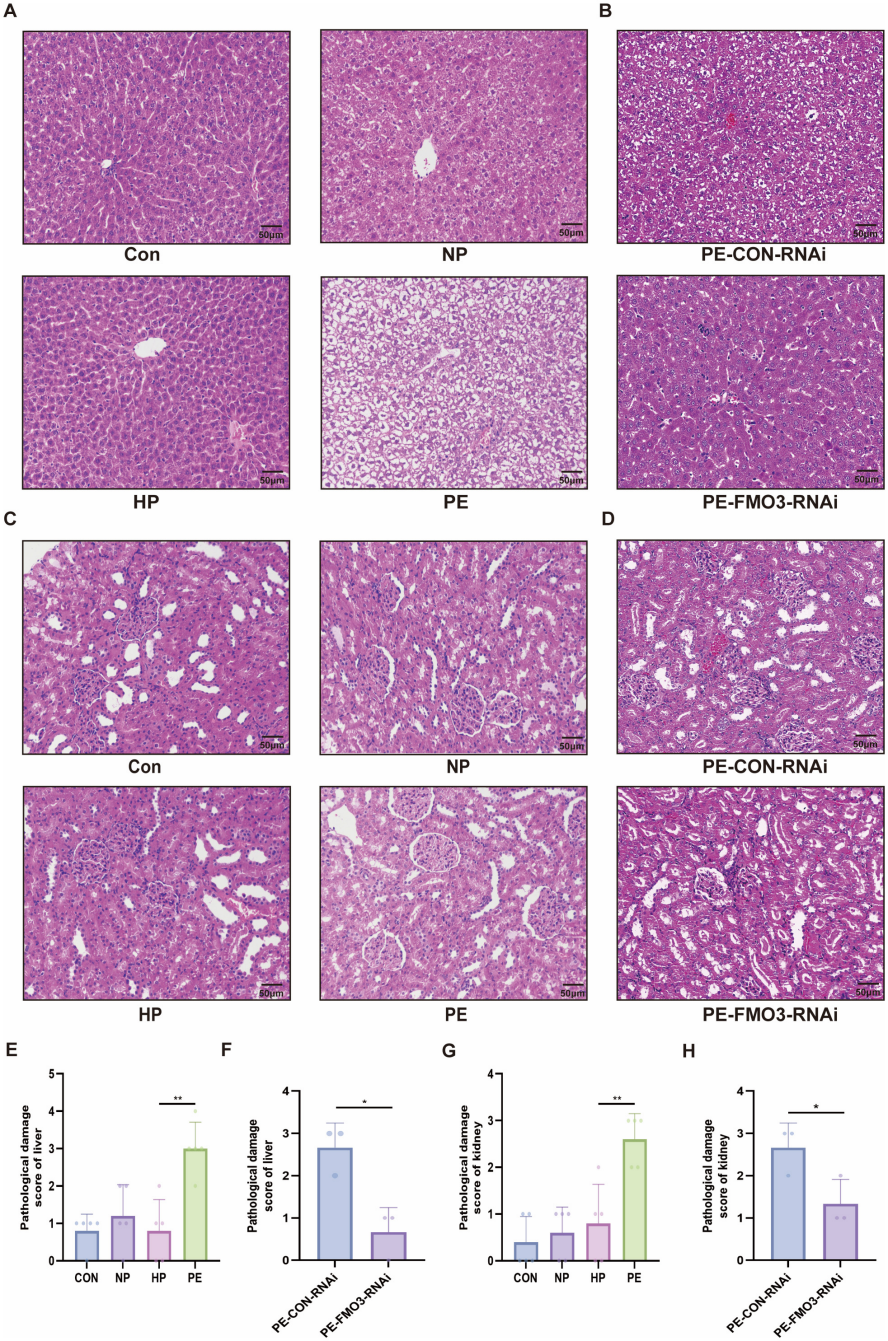
Histopathological examination of rat liver and kidney. **(A)** Sample images of liver sections stained with hematoxylin and eosin (H&E) for each fecal microbiota transplantation group. **(B)** Sample images of liver sections stained with hematoxylin and eosin (H&E) for PE-CON-RNAi and PE-FMO3-RNAi group. **(C)** Illustrative examples of kidney tissue sections stained with hematoxylin and eosin (H&E) for each fecal microbiota transplantation group. **(D)** Sample images of kidney sections stained with hematoxylin and eosin (H&E) for PE-CON-RNAi and PE-FMO3-RNAi group. **(E–H)** Blinded pathological damage score of liver and kidney were analysis by two expert pathologists (PS: *p < 0.05, **p < 0.01, all the results based on at least three repetitions, data of multiple groups were analyzed by one-way ANOVA. *T* tests were used to compare the differences between two groups).

It is well-known that preeclampsia can cause damage to various organs such as liver and kidney and FMO3 is mainly expressed in the liver. We further explored the pathological conditions of liver and kidney in each group. Compared with Con, HP and NP groups, the pathological damage of liver and kidney in PE group was more obvious (Figures 4A, C). In the PE group, hepatic plate structure was disordered, and some areas of the hepatic plate structure were unclear and collapsed. The cytoplasm became translucent due to edema (Figure 4A). Compared with Con, HP and NP groups, the kidney cells were slightly edematous in PE group. A small amount of congestion was seen within the glomeruli (Figure 4C). The pathological damage of the liver and kidney in the PE-CON-RNAi group was similar to that in the PE group, but it was found to be alleviated in the PE-FMO3-RNAi group (Figures 4B, D).

We also quantitatively analyzed the above results. Blinded pathological damage score of liver and kidney were analysis by two expert pathologists. Pathological damage score was significantly increased in the PE group (Figures 4E, G), but decreased after FMO3 knockdown (Figures 4F, H). It can be seen from the pathological qualitative and quantitative analyses of liver and kidney in each group that the transplantation of gut microbiota in preeclampsia rats can lead to damages of liver and kidney, and this effect can be alleviated after FMO3 knockdown.

### 3.5 PE-fecal microbiota transplantation in SD rats can lead to inflammation which were alleviated by FMO3 knockdown

**Figure 5:**
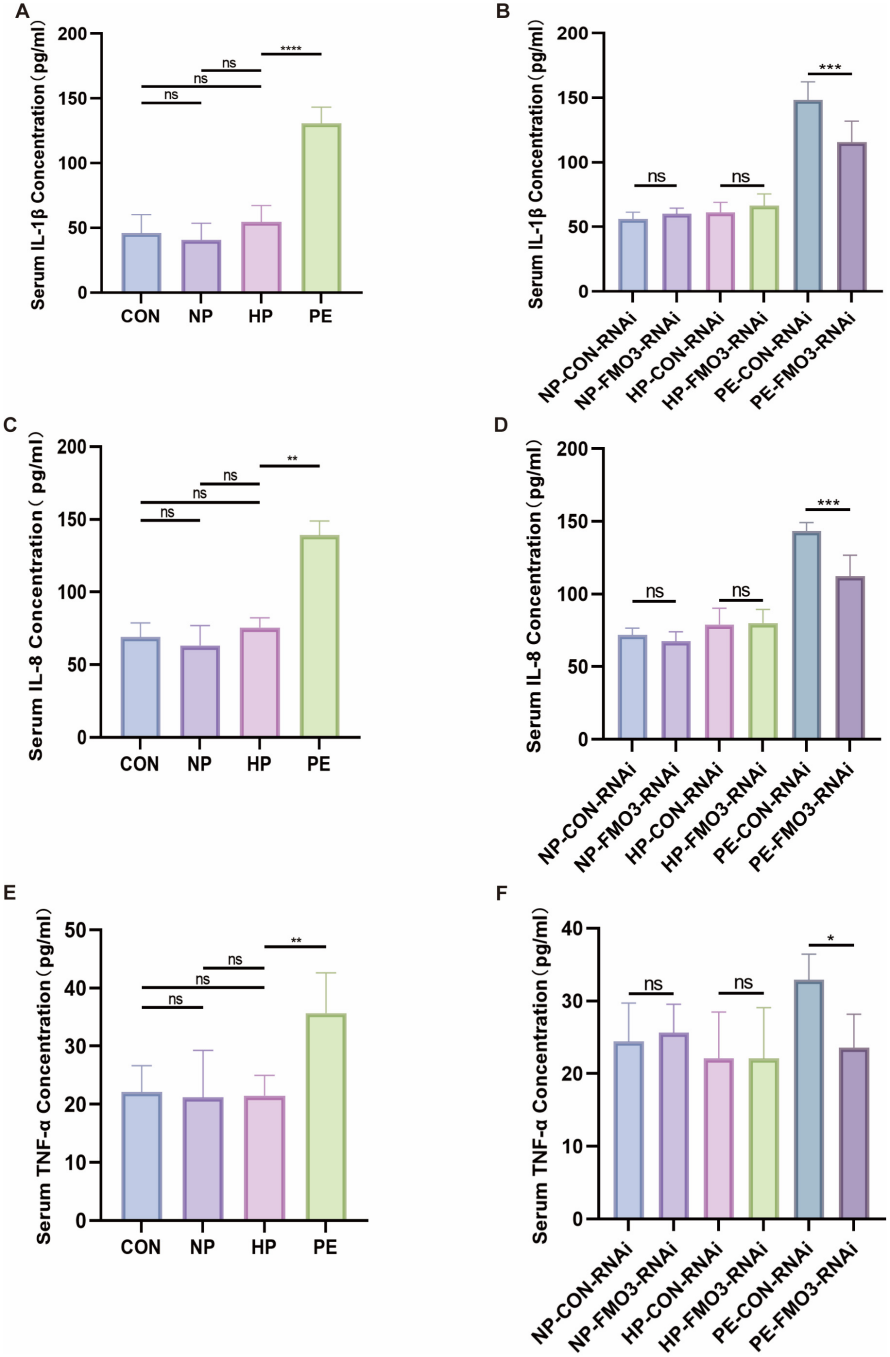
Serum inflammatory factor levels in maternal rats. **(A)** Serum levels of IL-1β of Con, NP and, groups. **(B)** Serum IL-1β level in each group after injection of FMO3-RNAi adenovirus. Serum levels of IL-8 across different groups. **(C)** TNF-α concentration in serum for each group. **(D)** Serum TNF-α concentration in each group following the administration of FMO3-RNAi adenovirus. **(E)** Concentration of IL-8 in serum for each group. **(F)** Concentration of IL-8 in serum for each group following FMO3-RNAi adenovirus administration (PS: *p < 0.05, **p < 0.01, ***p < 0.001, ****p < 0.0001, all the results based on at least three repetitions).

Previous studies have shown that the imbalance of gut microbiota can cause systemic inflammatory response (Hrncir, 2022), so we further explore the association between inflammatory factors and our findings. The IL-1β concentrations in the CON, NP, HP, and PE groups were (45.90 ± 14.36), (40.68 ± 12.86), (54.56 ± 12.70), and (130.83 ± 12.47) pg/mL, respectively. IL-8 levels, respectively (69.00 ± 9.83), (63.25 ± 13.73), (75.51 ± 6.81), and (139.15 ± 9.83) ng/L, TNF-α levels, respectively (22.10 ± 4.53), (21.21 ± 8.07), (21.49–3.48), and (35.68 ± 6.91) pg/mL. In comparison to the Con group, the PE group exhibited markedly elevated levels of IL-1, IL-8, and TNF-α, whereas the NP and HP groups showed no notable differences from the Con group (Figures 5A, C, E).

The IL-1β concentrations in the NP-CON-RNAi, NP-FMO3-RNAi, HP-CON-RNAi, HP-FMO3-RNAi, PE-CON-RNAi and PE-FMO3-RNAi groups were (56.18 ± 5.28), (60.05 ± 4.50), (61.28 ± 7.86), (66.62 ± 8.98), (148.46 ± 14.00), and (115.77 ± 16.17) pg/mL. IL-8 levels were, respectively (71.99 ± 4.62), (67.52 ± 6.59), (79.05 ± 11.19), (80.04 ± 9.46), (143.38 ± 5.94) and (112.34 ± 14.40) ng/L, TNF-α levels were, respectively (24.43 ± 5.29), (25.63 ± 3.91), (22.10 ± 6.39), (22.07 ± 7.02), (32.90 ± 3.54), and (23.58 ± 4.62) pg/mL. In comparison to the PE-CON-RNAi group, the PE-FMO3-RNAi group exhibited reduced levels of IL-1β, IL-8, and TNF-α which showed that that these inflammatory factors can be partially reversed by knocking down FMO3 (Figures 5B, D, F). All results showed that FMO3 knockdown may alleviate the inflammation in PE-FMT rats and FMO3 may be involved in PE pathological process via inflammation.

### 3.6 Analysis of the FMT bacterial abundance and changes in bacterial composition and function of PE-FMT after FMO3 knockdown

**Figure 6:**
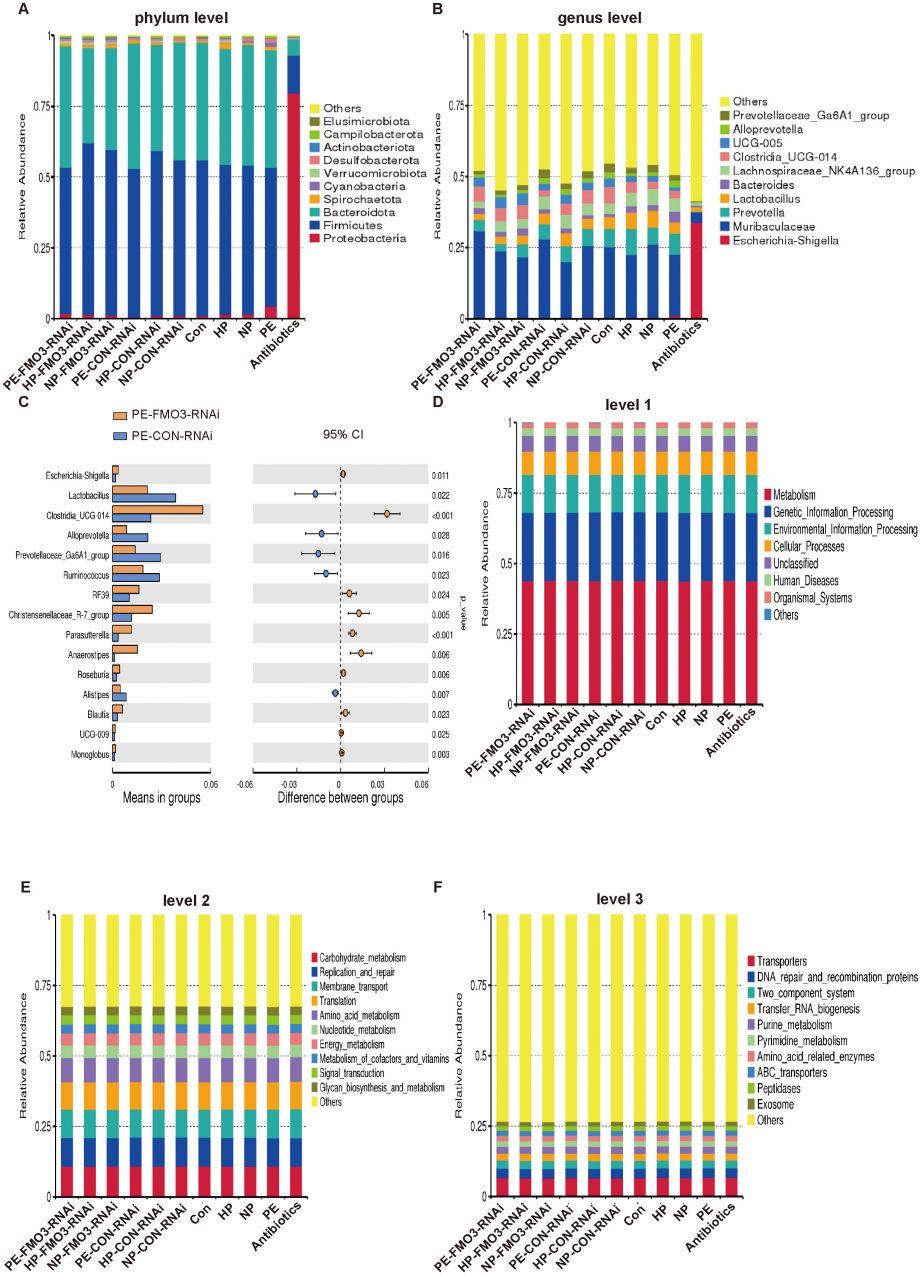
Rat gut microbiota changes. **(A, B)** The abundance of gut microbiota at phylum and genus levels in different groups. **(C)** Gut microbiota composition varied between the PE-FMO3-RNAi and PE-CON-RNAi groups. **(D–F)** Tax4Fun functional annotation relative abundance bar plot. Analysis the functional differences between different groups by Kruskal-Wallis test with Dunn's *post-hoc* test for multiple group comparisons.

In order to better explore how the transplantation of gut microbiota composition work in above results, we sequenced and analyzed the gut microbiota of each group. Firstly, we found that the antibiotic treatment group exists significant difference (*p* < 0.0001) of gut microbiota alpha diversity and the other no significant differences between groups (Figures S1H–M), which proved that our antibiotic depletion pretreatment had achieved the desired effect. According to the abundance of gut microbiota, it was divided into different phylums and genera. In each group, the top four phyla with the greatest relative presence included Proteobacteria, Firmicutes, Bacteroidota, Spirochaetota (Figure 6A). The top four genera with the greatest relative abundance included Escherichia-Shigella, Muribaculaceae, Prevotella, Lactobacillus (Figure 6B). We speculate that these phylums and genera may play an important role in the association between gut microbiota and preeclampsia.

Subsequently, we analyzed and compared the differences in gut microbiota between the PE-FMO3-RNAi and PE-CON-RNAi group in detail. Figure 6C illustrated that the intestinal microbiota composition varied between the PE-FMO3-RNAi and PE-CON-RNAi groups. The Wilcoxon rank-sum test was employed to analyze the variation in gut microbiota between the PE-FMO3-RNAi and PE-CON-RNAi group. The levels of Clostridia_UCG-014 and Parasutterella were notably elevated in the PE-FMO3-RNAi group compared to the PE-CON-RNAi group (*p* < 0.001). We speculate that FMO3 may play an important role in the association between gut microbiota and preeclampsia via those gut microbiota.

We further analyzed the main enrichment functions of gut microbiota between different groups via Tax4Fun. At level 1, the two main functions of the most common gut microbiota among groups are Metabolism and Genetic Information Processing (Figure 6D). At level 2, the main two predominant functions of gut microbiota across various groups were Carbohydrate metabolism and Replication and repair (Figure 6E). In various groups, the main two predominant functions of gut microbiota at level 3 included Transporters and DNA repair and recombination proteins (Figure 6F).

**Figure 7:**
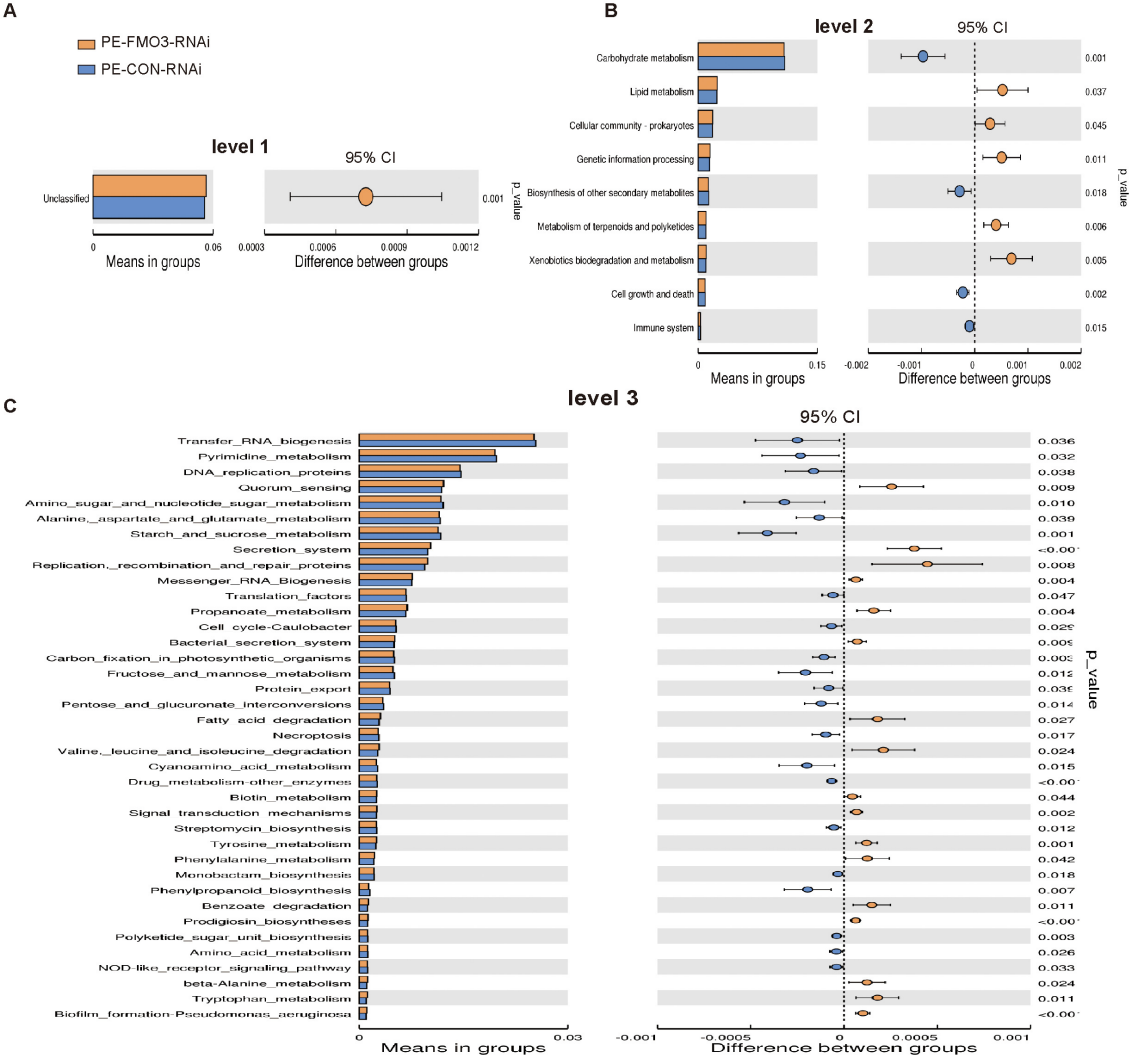
Rat gut microbiota function changes between the PE-FMO3-RNAi and PE-CON-RNAi groups. **(A–C)** Tax4Fun functional annotations between the PE-FMO3-RNAi and PE-CON-RNAi groups in different level. Analysis the functional differences between different groups by Kruskal-Wallis test with Dunn's *post-hoc* test for multiple group comparisons.

Furthermore, we compared the differences between the predicted gut microbiota functions between the PE-FMO3-RNAi and PE-CON-RNAi group in detail. The results showed that only Unclassified was different between the two groups at level 1 (*p* = 0.001, Figure 7A). At level 2, the top two variations were observed between the two groups in Carbohydrate metabolism (*p* = 0.001) and Cell growth and death (*p* = 0.002) between the two groups (Figure 7B). At level 3, variations were observed mainly in Secretion system, Drug metabolism-other enzymes, Biofilm formation-Pseudomonas aeruginosa and Prodigiosin biosynthesis (*p* < 0.001) between the two groups (Figure 7C). All the results may indirectly suggest that the gut microbiota may affect the pathogenesis of preeclampsia through the above phylums and genera, and FMO3 knockdown may affect PE process by affecting the specific functions of certain specific bacteria. But further research is still needed.

### 3.7 Functional prediction of gut microbiota associated with PE and regulation of trophoblast and uterine artery endothelial cell function by IL-8

**Figure 8:**
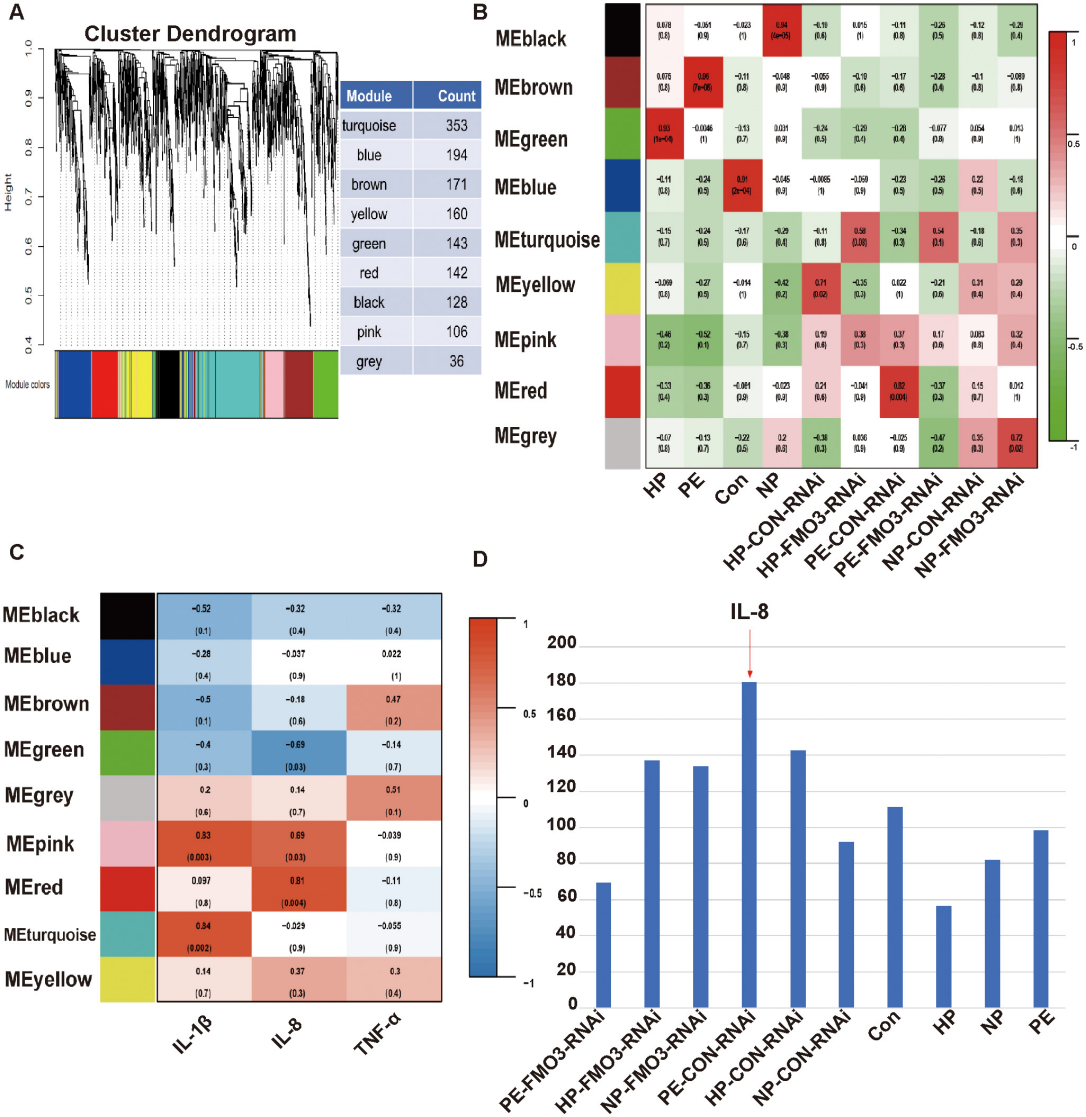
Functional prediction of gut microbiota associated with PE. **(A)** Cluster analysis of different bacterial groups and each cluster is represented by a color. **(B)** Heat map representing the association between different flora clustering and experimental groups. **(C)** Heat map representing the association between different flora clustering and inflammatory factors. **(D)** Bar graph of correlation between experimental groups and IL-8 phenotype.

Further, in order to explore the relationship between gut microbiota, PE and inflammation, we clustered the gut microbiota and analyzed its correlation with different phenotypes and inflammatory factors. According to the content of gut microbiota, different bacteria were clustered and named with different color (Figure 8A), and the correlation between different microbiota clusters and related phenotypes were analyzed (Figure 8B). The bacterial flora involved in the Red cluster was highly positively correlated with the PE-CON-RNAi phenotype. Furthermore, we found that the Red cluster was also highly related with IL-8 (Figure 8C). This suggests that IL-8 may be an important inflammatory factor involved in the pathological changes of PE-FMT group.

By correlation analysis of co-expression networks, the core flora in different bacterial groups was found. Based on the screening of the top 10 hub bacteria and the OTU identification, Lachnospiraceae_NK4A136_group might be the central bacterium in the Red flora module (Figure S3). The microbiota involved in the Red flora module was highly positively correlated with IL-8 phenotype and PE-CON-RNAi phenotype, and the comparison of the bar chart showed that PE-CON-RNAi and IL-8 had a certain synergistic effect (Figure 8D).

**Figure 9:**
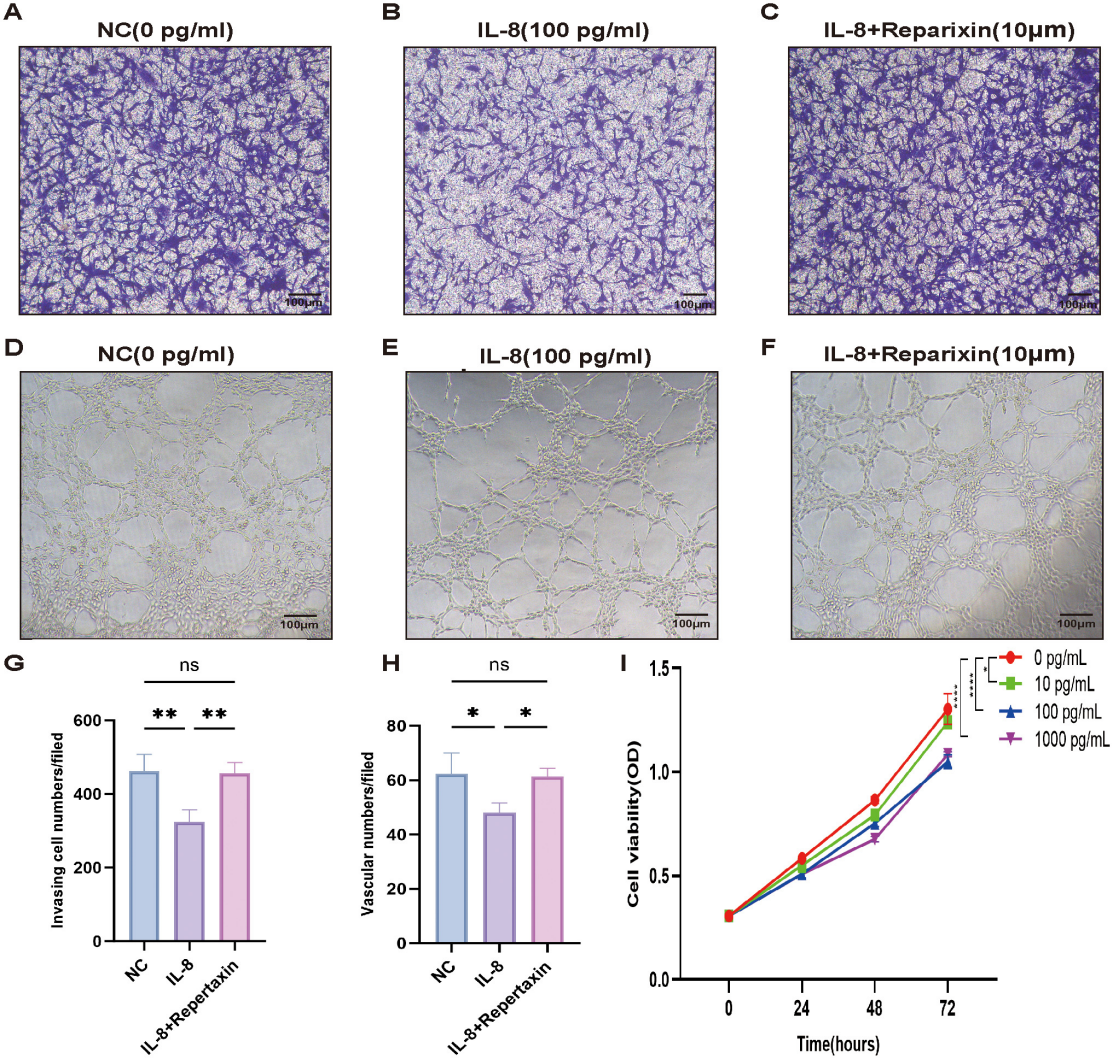
Regulation of trophoblast and uterine artery endothelial cell function by IL-8. **(A–C, G)** Examples showing the quantity of invasive cells after IL-8 (100 pg/mL) and reparixin (10 μm) treatment. **(D–F, H)** Representative images of tubes formed after IL-8 (100 pg/mL) and reparixin (10 μm) treatment. **(I)** Cell growth was assessed using the CCK8 assay at intervals of 0, 24, 48, and 72 h with varying IL-8 concentrations (PS: *p < 0.05, **p < 0.01, ****p < 0.0001, all the results based on at least three repetitions).

Later, CCK8 and Transwell tests were employed to examine how IL-8 influences the growth and migration of trophoblast cells. Upon IL-8 stimulation, the invasive ability of HTR8/sVneo cells was impaired, and the number of cells invaded in the 100 pg/mL group was less than that in the 0 pg/mL group (Figures 9A, B). To further test the hypothesis that IL-8 is related to poor placental angiogenesis, we used HUVECs cells co-cultured with 0 and 100 pg/mL IL-8 for 48 h and found that the amount of angiogenesis in the 100 pg/mL group was reduced compared with the 0 pg/mL group (Figures 9D, E). In addition, this situation can be rescued by IL-8 receptor antagonist reparixin (Figures 9C, F–H). Moreover, as IL-8 levels rose, the growth of HTR8/sVneo cells markedly declined (Figure 9I). This revealed that the increase of IL-8 in PE-FMT may lead to preeclampsia-like symptoms by affecting placental trophoblast cell viability, invasion ability and placental angiogenesis.

## 4 Discussion

This study demonstrates that fecal microbiota transplantation from PE rats induces preeclampsia-like symptoms, including hypertension, renal impairment, and systemic inflammation characterized by elevated IL-1β, IL-8, and TNF-α. Previous studies had shown that intestinal microbiota were tightly associated with PE (Beale et al., 2019; Yang, 2022). Furthermore, inflammatory factors such as IL-6, IL-1β, and TNF-α were also proved associated with imbalanced intestinal microbiota (Giugliano et al., 2025). Probiotics may improve vascular endothelial function, lower blood pressure and inflammation in the body (Chen et al., 2022). Our findings align with clinical observations linking gut dysbiosis to PE pathogenesis and extend them by establishing a direct causal relationship in an animal model.

FMO3 was an enzyme facilitates the N-oxygenation of TMA, producing non-smelly trimethylamine N-oxide (TMAO; Chen et al., 2019; Iglesias-Carres et al., 2023; Yang et al., 2022; Gatarek and Kaluzna-Czaplinska, 2021). In our study, the role of FMO3 in PE was examined through the use of FMO3-RNAi, which led to a notable reduction in systolic blood pressure and urine protein in PE model rats. At same time, fetal outcomes and liver and kidney pathological damage have also been significantly improved. This finding underscores the importance of FMO3 in the pathophysiology of PE, potentially through its involvement in inflammatory pathways. Increased concentrations of inflammatory cytokines (IL-1β, IL-8, and TNF-α) were detected in PE rats, but these levels significantly decreased after FMO3 knockdown, indicating that FMO3 might influence inflammatory reactions. Additionally, the effect of FMO3 on gut microbiota composition and function was apparent from the alterations in bacterial species and metabolic pathways following FMO3 knockdown. The increase in Escherichia-Shigella and decrease in Lactobacillus in FMO3-RNAi rats indicate that FMO3 may influence gut microbiota dynamics, which could have downstream effects on host metabolism and immune responses. These findings align with previous studies that have shown FM3's involvement in metabolic processes beyond TMAO production, including its role in the urea cycle and interaction with other metabolic enzymes (Luo et al., 2024).

We also found that the Proteobacteria, Firmicutes, Bacteroidota, Spirochaetota were the top four genera with the greatest relative abundance. In 2019, Wang et al. examined the fecal gut microbiota of patients with PE and found a significant differences in the abundance of Firmicutes (51.64% PE vs. 59.62% Control, *P* < 0.05), Bacteroidetes (40.51% PE vs. 34.81% Control, *P* < 0.05), Proteobacteria (4.51% PE vs. 2.56% Control, *P* < 0.05), and Actinobacteria compared to their healthy counterparts (Yong et al., 2022). In 2025, Li et al. reported that an imbalance of Bacteroidetes, Firmicutes, and Proteobacteria between PE, severe PE, non-severe PE and healthy pregnant women which is consistent with our and wang's findings (Li et al., 2025). Moreover, we found that Clostridia_UCG-014 and Parasutterella were notably elevated in the PE-FMO3-RNAi (*p* < 0.001) which were proved to be significantly enriched in the patients of high TMAO (Wu et al., 2020). At the same time, the levels of FMO3 and TMAO in the PE-CON-RNAi group were significantly increased. After knocking down FMO3, the symptoms of PE-CON-RNAi were alleviated and the level of TMAO was significantly decreased. IL-8 and PE phenotypes were proved significantly correlated with these hub bacteria. IL-8 can significantly affect the activity and invasion of placental trophoblast cells and the angiogenesis of endothelial cells *in vitro*. Furthermore, the rescue of trophoblast and endothelial cell function upon IL-8 receptor blockade provides more direct evidence for the causal role of IL-8 in mediating these detrimental effects. These results suggest that FMO3-mediated TMAO metabolism may affect the IL-8-meiated inflammatory response in the pathogenesis of preeclampsia. The gut microbiota that produces TMAO may be the key flora responsible for the occurrence and development of PE.

Although the discoveries are important, it's essential to recognize the various limitations of this research. First, the study relied primarily on animal models and cell experiments and did not incorporate clinical trials, which limits the direct applicability of the findings to human health. In addition, the sample size was relatively small, which may affect the statistical power and generalizability of the findings. Upcoming research ought to tackle these shortcomings by incorporating more extensive sample groups and conducting clinical experiments. At the same time, it is still necessary to further explore which gut microbiota affects preeclampsia through what functions.

Secondly, although a number of studies have shown that L-NAME can successfully establish animal models of PE which can including elevated blood pressure and proteinuria, renal function damage, typical renal pathology such as glomerular endothelial disease, liver function damage and focal necrosis in the surrounding area of hepatic lobules, placental inflammatory cell infiltration, fibrinoid necrosis and fibrosis (Shu et al., 2018; Soobryan et al., 2017; Selivanova et al., 2021; Yıldırım et al., 2014) which has been reported to be most consistent with the human PE phenotype (Shu et al., 2018), the occurrence and development of PE is a complex process. At present, no animal model can completely replicate the natural process of the disease. Because the experimental design requires long-term and multiple gut microbiota transplantation, we used this model instead of PE pregnant women. This limits the universality of our experimental results. However, the transplantation of gut microbiota in rats is still helpful for us to further explore the relationship between gut microbiota and PE. Next, we will further explore the role of gut microbiota in the pathophysiological state of pregnant women with PE.

In summary, the results showed that fecal microbiota transplantation from rats with PE led to increased blood pressure, and notable variations were found in the expression of FMO3 protein and mRNA levels of target genes among the various treatment groups. Microscopic examination revealed significant structural alterations in liver and kidney tissues, while ELISA tests demonstrated considerable differences in IL-1β, IL-8, and TNF-α concentrations, implying modified inflammatory reactions. Cell function assays further confirmed the impact of IL-8 on cell viability, invasion, and tube formation. These findings establish an integrated pathway connecting gut microbiota dysbiosis, FMO3 upregulation, inflammatory activation, and the clinical manifestations of PE, laying the groundwork for future studies to investigate treatments aimed at gut microbiota to manage PE. This study successfully established a rat model of gut microbiota dysbiosis induced by oral antibiotics and demonstrated that fecal microbiota transplantation from different sources significantly affects pregnancy-induced hypertension and inflammatory responses.

## Data Availability

The data presented in this study are publicly available. The data can be found at: https://ngdc.cncb.ac.cn/, accession PRJCA045176. Further inquiries can be directed to the corresponding authors.
