# Supplementary material for: The role of gut microbiota imbalance in preeclampsia pathogenesis: insights into FMO3-mediated inflammatory mechanisms
Source: Front Microbiol. 2025 Dec 12;16:1682007. doi: 10.3389/fmicb.2025.1682007 (PMC12742473; doi:10.3389/fmicb.2025.1682007)
Supplement: Supplementary file 1 [file Supplementary_file_1.docx]

**Supplementary Material**

# **Supplementary Figures and Tables**

| **Group** | **Liver Tissue Appearance** | **Kidney Tissue Appearance** |
| --- | --- | --- |
| Con group | The liver cells were organized in a single layer, radiating outward from the central vein. The hepatocytes cytoplasm was eosinophilic and abundant with uniformly sized nuclei. Some hepatocytes were binucleated and slightly edematous. A small amount of Kupffer cell proliferation and isolated inflammatory cell infiltration were observed in the hepatic sinusoids. | The renal tubules were closely arranged. The cells were cuboidal, with round/ovoid nuclei and regular arrangement. The basement membrane was intact, and there was no obvious edema and inflammatory cells in the stroma. There were no apparent abnormalities in the glomeruli. |
| NP group | The liver cells were organized in a single layer, radiating around the central vein. The hepatocyte cytoplasm appeared eosinophilic, and the cells were marginally larger compared to typical hepatocytes. Some hepatocytes were binucleated. The cytoplasm was partially transparent and individual eosinophilic necrosis was seen. The cell volume was increased, but slightly smaller than in the PE group. A small amount of Kupffer cell proliferation was seen in the hepatic sinusoids. | The renal tubules were still closely arranged. Some cells were slightly edematous. The cells were cuboidal, with round/ovoid nuclei and regular arrangement. The basement membrane was largely intact. Exfoliation of the epithelial epithelium of individual tubules resulted in the exposure of the basement membrane and exfoliation of individual brush borders. There was no obvious edema and inflammatory cells in the interstitium, and there was mild congestion of the interstitial vessels. There were no apparent abnormalities in the glomeruli. |
| HP group | The liver cells were organized in a single layer, radiating around the central vein. The hepatocyte cytoplasm appeared eosinophilic, featuring plentiful cytoplasm and consistently sized nuclei. Some hepatocytes were binucleated. A small amount of Kupffer cell proliferation and isolated inflammatory cell infiltration were observed in the hepatic sinusoids. | The renal tubules were still closely arranged. A few cells were slightly edematous. The cells were cuboidal, with round/ovoid nuclei and regular arrangement. The basement membrane was intact, and there was no obvious edema and inflammatory cells in the stroma. There was mild congestion of the interstitial vessels. There were no apparent abnormalities in the glomeruli. |
| PE group | Hepatic plate structure was disordered, and some areas of the hepatic plate structure were unclear and collapsed. Most of the cells were enlarged and showing obvious edema and ballooning. The cytoplasm became translucent due to edema, Mallory bodies were seen in some cytoplasm, the cytoplasm was slightly basophilic and some nuclei were pyknotic. A minor increase in Kupffer cell growth and slight lymphocyte infiltration were noted in the liver sinusoids. | The renal tubules were still tightly arranged. A few cells were slightly edematous. The cells were cuboidal, with round/ovoid nuclei and regular arrangement. The basement membrane was intact, and there was no obvious edema and inflammatory cells in the stroma. A small amount of congestion was seen within the glomeruli, but the rest were unremarkable. |
| PE-CON-RNAi group | The overall structure of liver tissue was abnormal, the arrangement of liver cells was irregular, the structure of liver cells was loose, more liver cells were loose and edematous, some of them were edematous to vacuolar degeneration, the structure of liver sinus was unclear, and no obvious inflammatory cell infiltration was found in the tissue. | The tissue showed more slight expansion of renal tubules, and the epithelial cells were loose and edematous. No fibrous connective tissue hyperplasia was found in the interstitial tissue, and no obvious inflammatory cell infiltration was found. |
| PE-FMO3-RNAi group | The arrangement of liver cells was regular. The structure of liver cells was full. There was no loose edema and necrosis of liver cells. The structure of central vein was clear. The hepatic sinus was radially arranged along the central vein. There was no obvious congestion and dilatation in the hepatic sinus, and a small amount of inflammatory cell infiltration was observed. | No obvious atrophy and lobulation necrosis of glomerulus was observed. There was no fibrous connective tissue hyperplasia in the interstitial tissue, and no inflammatory cell infiltration was observed. |

**Table.1** Histopathological Observations on Liver and Kidney Tissues among Multiple Groups


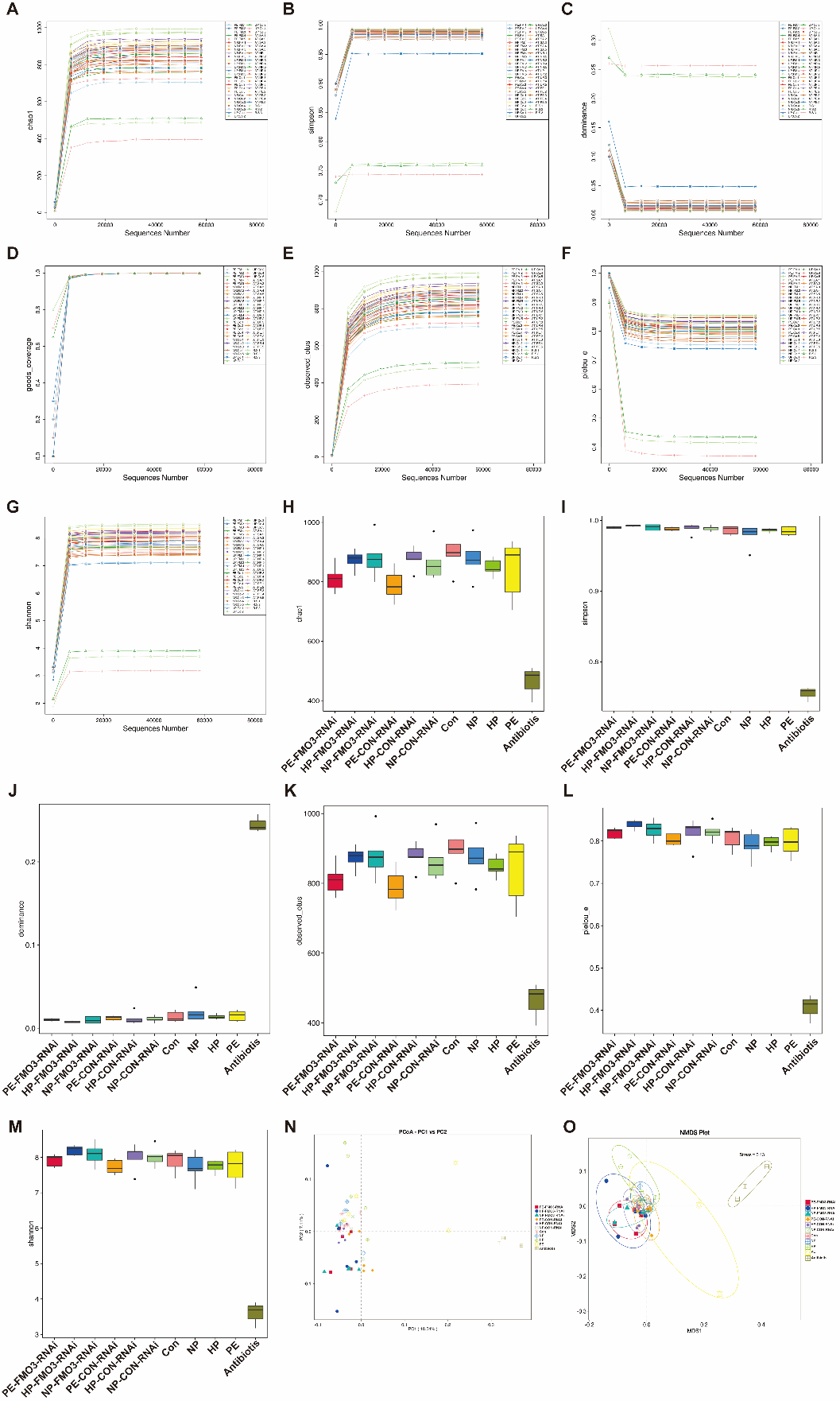


**Figure.1** Alpha and Beta diversity analysis of gut microbiota

(A-G) Species dilution curves were analyzed using chao1, Simpson, dominance, Good's coverage, Observed otus, Pielou e and Shannon methods. (H-M) Alpha diversity index were analyzed via chao1, Simpson, dominance, observed otus and Pielou e and Shannon. (N) Beta diversity was analyzed via principal coordinate analysis (PCoA). (O) Without measuring multi-dimensional calibration method (NMDS) assessment evaluation results of beta diversity stress.

In order to verify whether our sequencing data can reliably reflect the diversity in the sample. Species dilution curves were analyzed using chao1, Simpson, dominance, Good's coverage, Observed otus, Pielou e and Shannon methods (Fig. S1A-G). The findings revealed that with an increase in sequencing data, the curve levels off, suggesting that the data volume becomes adequate, and additional data will not significantly affect the alpha diversity index.

Based on unweighted Unifrac distance analysis, using the principal coordinate analysis (PCoA) to evaluate microbial beta diversity shows: PCo1 and PCo2 contribution rate were 18.54% and 7.11% respectively (Fig. S1N). Without measuring multi-dimensional calibration method (NMDS) assessment evaluation results showed that microbial beta diversity Stress = 0.13, indicate that NMDS can accurately reflect the extent to which the differences between samples, each one in figure represents the point has obvious way of clustering (Fig. S1O). All the results shows that our sequencing results are reliable.


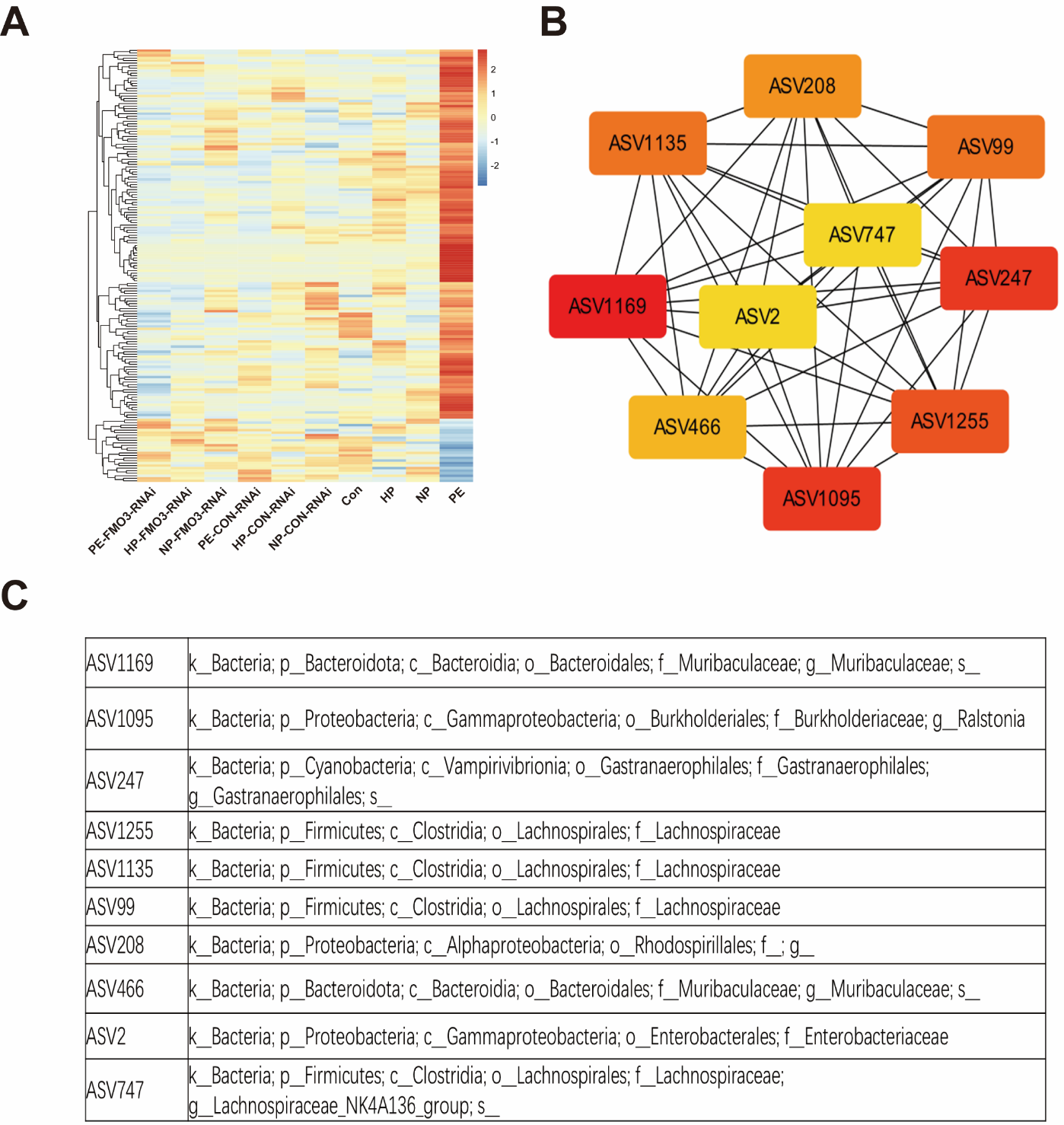


**Figure.2** The top10 hub flora involved in the positive correlation between the Brown module and the PE phenotype

(From yellow to red, the deeper the color, the stronger the correlation.)

Based on the screening of the top 10 hub bacteria and the OTU identification, Muribaculaceae might be the central bacterium in the Brown flora module (Fig. S2).


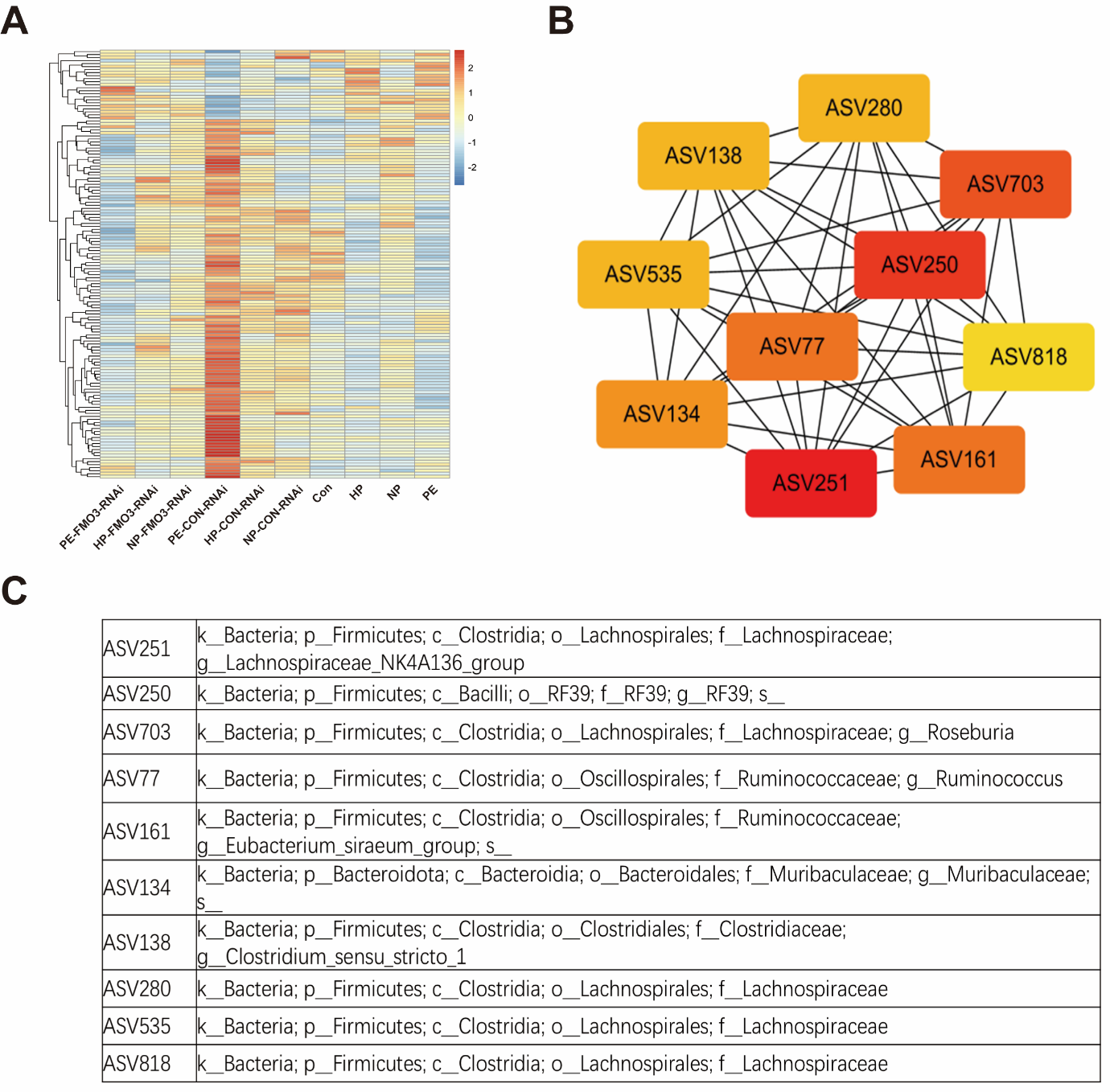


**Figure.3** The top10 hub flora involved in the positive correlation between the Red module and the PE-CON-RNAi phenotype

(From yellow to red, the deeper the color, the stronger the correlation.)
